# Further Development and Testing of a Compact Wind Tunnel for Exposing Mosquitoes to Formulated Insecticide Products

**DOI:** 10.3390/insects16111180

**Published:** 2025-11-19

**Authors:** Stephanie Richards, Sinan Sousan, Qiang Wu, Will Murray, Emma Rush, Raven Slade, Paul Jones, Avian White, Naia Braxton

**Affiliations:** 1 Department of Health Education and Promotion, College of Health and Human Performance, East Carolina University, Greenville, NC 27858, USA; murrayjo20@students.ecu.edu (W.M.); rushem21@students.ecu.edu (E.R.); slader20@students.ecu.edu (R.S.); jonespa22@students.ecu.edu (P.J.); whiteav15@ecu.edu (A.W.); 2 Department of Public Health, Brody School of Medicine, East Carolina University, Greenville, NC 27858, USA; sousans18@ecu.edu (S.S.); wuq@ecu.edu (Q.W.); 3 NC Agromedicine Institute, East Carolina University, Greenville, NC 27858, USA; 4 Center for Human Health and the Environment, NC State University, Raleigh, NC 27695, USA; 5 Department of Biological and Forensic Sciences, Fayetteville State University, Fayetteville, NC 28301, USA; braxtonnaia@gmail.com

**Keywords:** insecticide resistance, mosquito control, insecticide efficacy

## Abstract

Insecticide resistance is increasing globally, and tools must be developed to combat this issue facing mosquito control programs that protect public health and inform operational decisions. Field trials to assess insecticide formulated products (FPs) are logistically demanding and weather-dependent and the Centers for Disease Control and Prevention bottle bioassays are optimized to test technical active ingredient (AI) residue, not aerosolized FP. Here, the methodological gap between AI and FP efficacy testing is addressed. The current study assessed the knockdown/mortality of laboratory and wild populations of *Aedes albopictus* and *Culex pipiens/quinquefasciatus* to four FPs (Biomist^®^, Duet^®^, AquaDuet^®^, ReMoa Tri^®^) in wind tunnel experiments. The number of FP droplets on mosquitoes was analyzed. Regression analyses showed that droplet counts on mosquitoes were significantly (*p* < 0.05) related to mosquito mortality for some FPs. The wild *Culex* population was resistant to all FPs in the wind tunnel. Here, when wind tunnel experiments resulted in a relatively low mortality rate (<90%), this indicates mosquito resistance to the FP. In these situations, a field trial would likely not achieve good results and may not be necessary. Alternatively, when wind tunnel experiments resulted in a nearly perfect mortality rate, a confirmatory field trial could be conducted, if needed.

## Simple Summary

Insecticide resistance is a global issue that mosquito control and other programs are facing that must be addressed to protect public health. Targeted control using the most efficacious insecticide products will help reduce insecticide resistance. A novel compact wind tunnel was developed to quickly assess product efficacy against mosquitoes in a laboratory. Oil- and water-based products were tested against laboratory and field mosquito populations and operational suggestions are provided. The wind tunnel can be used as a screening step to inform field trials and/or when it is not possible to conduct weather-dependent and labor-intensive field trials.

## 1. Introduction

Billions of people are at risk and approximately 700,000 deaths occur each year worldwide from mosquito-borne diseases [1]. West Nile encephalitis (WNE) is the most prevalent locally acquired mosquito-borne disease in the United States (US) and is caused by mosquito-borne transmission of West Nile virus (WNV) [2]. The US experienced 59,141 cases of WNV infections, resulting in >27,000 hospitalizations and ≈2958 deaths during 1999–2023 [2]. In 2024 alone, 1466 cases were reported across 49 states, reaffirming WNV as the most prevalent locally transmitted pathogen by mosquitoes [2]. Furthermore, despite being primarily nuisance biters, floodwater and other mosquitoes can impede emergency response after natural disasters such as hurricanes [3]. Therefore, mosquito control strategies must be optimized to address a variety of mosquito species and populations.

Insecticide resistance (IR) is increasing globally; between 2010 and 2020, 78 of 88 malaria-endemic countries reported resistance to at least one insecticide in malaria vectors [4]. Even in some countries where resistance to pyrethroids has been documented, this class of insecticide continues to be used by mosquito control programs (MCPs) [5]. The lack of alternatives in available tools, operational manpower, and strategic bandwidth has led to the continued use of insecticide classes such as pyrethroids, despite their declining efficacy.

Current methods for assessing insecticide formulated product (FP) efficacy for mosquito control can be cumbersome (e.g., large-scale wind tunnels) and/or require logistically challenging, time-consuming, expensive, and weather-dependent field trials with multiple personnel working at dusk/dawn periods [1,6,7,8]. One study constructed a large-scale (2.4 m long) wind tunnel (i.e., dispersion tunnel) to test how wind speed affected droplet deposition on cages [9]. The same study concluded that higher wind speeds would be needed to move smaller droplets through mesh cages and larger droplets are most likely to be filtered out through the mesh of mosquito cages. Another large-scale (≈3.6 m long) wind tunnel device was used to expose flies and cockroaches to FP for 5 s [10]. The same study did not transfer insects from wind tunnel cages to clean cages after treatment, as they observed negligible differences in mortality, which reduced the handling of insects. In another study, a 1.2 m wind tunnel was used to expose mosquitoes to FP (Duet^®^ contains prallethrin which has excitation properties) or components of Duet^®^ (i.e., synergized prallethrin, sumethrin) using an airbrush [11]. The same study recorded videos of mosquitoes during treatments to evaluate activity levels and counted/measured the size of the droplets landing on different body parts of the mosquitoes. Droplets were measured on dissected mosquito body parts mounted on slides using a compound microscope (no fluorescent dye was used) [11]. Mosquitoes exposed to Duet^®^ or prallethrin alone showed more movement than control mosquitoes and increased movement may resulted in a greater number of droplets landing on the mosquitoes [11]. Most droplets were found on the legs and wings and were 2.5–5 µm in diameter [11]. The same study showed no correlation between the volume of droplets (i.e., droplet size or dose) and the number of droplets, indicating that several small droplets might have the same effect as fewer large droplets. Others have shown that droplets with a diameter from 2 to 16 µm dispersed in an ultra-low volume (ULV) FP cloud are the most likely to contact flying mosquitoes, even if larger (e.g., 32 µm) droplets were found on slides in the field [12].

Many small- to medium-scale MCPs do not routinely conduct field trials or use other methods to evaluate FP, partially due to a lack of resources. There is currently a methodological gap between technical active ingredient (AI) testing with the Centers for Disease Control and Prevention (CDC) bottle bioassays and the operational knowledge of FP efficacy [8,13,14,15], which this study seeks to address. More work is needed to link IR data to operational outcomes [14,16]. Resistance to an AI shown in a laboratory bioassay does not necessarily indicate the failure of the FP containing that AI, since FPs may have additional ingredients (e.g., synergists) that enhance effectiveness. Ideally, efficacy testing of FPs should be routinely carried out on target mosquito populations to ensure efficacy and reduce unnecessary spraying of ineffective insecticides [17,18]. Furthermore, since there is widespread resistance to pyrethroids and AIs from other insecticide classes globally, increased testing of FPs is needed to inform operational response to protect public health from mosquito-borne diseases (e.g., dengue, malaria, West Nile encephalitis) and aid in the response after natural disasters such as hurricanes. If a local mosquito population is resistant to an FP, it might be possible to rotate to another class or type of FP; hence, knowledge of FP resistance/susceptibility enables MCPs to make evidence-based operational decisions [14]. Both susceptible and resistant mosquito populations should be evaluated in lab and/or field trials to approximate real-world conditions since IR is increasing globally [19]. Many MCPs do not have the resources for FP efficacy testing; therefore, there is a need for a practical, low-cost solution to rapidly test and identify effective FPs for local mosquito populations before large-scale implementation.

A novel compact wind tunnel prototype was developed that fits inside a small fume hood and proof-of-concept results have been reported [7]. Briefly, the previous method development study exposed *Aedes albopictus* (Skuse) and *Culex pipiens* (Linnaeus)/*quinquefasciatus* (Say) to Biomist^®^ 3 + 15 (hereafter, Biomist^®^) (FP; permethrin AI) or air (control) in field trial cages within the wind tunnel. Mosquito populations were successfully exposed to Biomist^®^ in the wind tunnel and showed 100% mortality in all tested populations, although only a 2 h time point was monitored in this pilot study [7]. Consequently, the objectives in the current work were to (1) develop a new and improved research-grade wind tunnel prototype and (2) assess the proof-of-concept that the laboratory wind tunnel data can be used as an FP screening tool to inform operational decisions for MCPs.

## 2. Materials and Methods

### 2.1. Compact Wind Tunnel Construction

**Figure 1:**
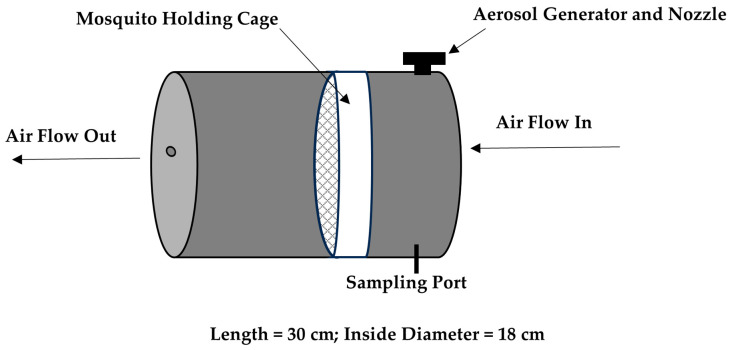
Wind tunnel prototype general diagram. The mosquito holding cage (white object) is placed inside the wind tunnel prior to insecticide aerosolization. Background air enters the inlet and exits the outlet of the chamber at 100 LPM.

A research-grade compact wind tunnel was constructed, incorporating improvements based on data from a previous prototype (Figure 1) [7]. Improvements include a commercial nozzle designed to optimize droplet sizes, dual valves to independently control air flow and liquid feed, dual filters to remove aerosol and possible volatile organic carbon, and dual gauges to independently control background air and nozzle air pressure. The main difference from the original wind tunnel model is the elimination of a Blaustein Atomizing Module (BLAM), a costly aerosol generator. Rather, the nozzle is equipped with a lower-cost commercial-based air atomizing body, fluid cap, and air cap (Spraying Systems, Co., Glendale Heights, IL, USA). Images of the wind tunnel model used here are shown (Figure 1). Lab-supplied air is filtered through a five-stage desiccant air dryer (Speedaire-2YNL6, Philadelphia, PA, USA) inside a small box that includes a High-Efficiency Particulate Air (HEPA) filter (Global Life Sciences Solutions 0.2 μm PTFE, Buckinghamshire, UK) open to the atmosphere to remove excess air and relieve pressure before entering the chamber. Air enters the inlet of the chamber at 100 L min^−1^ (LPM) using a mass flow controller (Alicat Scientific, Tucson, AZ, USA). A vacuum pump is used at the outlet (Welch-Ilmvac2585B-50, Niles, IL, USA), which is regulated by a control valve (SMC vacuum regulator, Yorba Linda, CA, USA). Wind tunnel assessments were carried out at ambient room temperature (22.5 °C) and humidity (61.6%).

### 2.2. Mosquito Populations and Mortality

Mosquito populations used here are listed in Table 1. North Carolina (NC) is in a hybrid zone for *Cx. pipiens* and *Cx. quinquefasciatus*, and hence these populations are referred to as *Cx. pipiens/quinquefasciatus*. The F_0_ generation mosquitoes were collected from the field as eggs, reared, and the adults were used in experiments. *Aedes albopictus* eggs were collected from the field using seed germination paper placed within a 0.5 L black plastic cup with tap water and retrieved after 5–7 d. For the Curry Court (Pitt County, NC, USA) *Culex pipiens/quinquefasciatus* population, egg rafts were collected from the field in black plastic water pans (52 × 38 × 13 cm) with hay infusion as an attractant. The F_2_ generation *Ae. albopictus* from March 9, 1764 Dr. NE (hereafter, March Drive) (Brunswick County, NC, USA) were collected from the field and propagated for two generations in the laboratory to increase sample size using established methods [20,21,22].

Female mosquitoes 4–6 d old were aspirated from colony cages and transferred to 15.2 cm diameter cardboard with mesh screen (Clarke Mosquito, St. Charles, IL, USA) (11–18 mosquitoes/cage; 3–4 replicate cages/group) for use in wind tunnel experiments. After FP exposure in the wind tunnel, mosquitoes from each group were transferred to separate clean 0.5 L cardboard cages, provided 20% sucrose, and placed in a 28 °C incubator with 14 h light:10 h dark. Knockdown was recorded at 2 h post-exposure and mortality was recorded at 24, 36, and/or 48 h post-exposure. The 36-h time point was used as the final mortality assessment time for mosquitoes exposed to Biomist^®^, Duet^®^, and AquaDuet^®^ (Clarke Mosquito Control, St. Charles, IL, USA), while the 48-h time point was used for ReMoa Tri^®^ (Valent BioSciences, Libertyville, IL, USA). Monitoring mortality at the later (48 h) time point is recommended for ReMoa Tri^®^ [18].

### 2.3. Aerosol Characterization (Droplet Size)

Prior to mosquito exposure, aerosols generated in wind tunnel assays were characterized and optimized following the manufacturer instructions using an Aerodynamic Particle Sizer (spectrometer) (APS 3321, TSI Incorporated, Shoreview, MN, USA) with probe inserted through a sample port located near the nozzle. Each FP was aerosolized for at least five minutes to determine droplet size (mass median diameter [MMD]).

In this study, the wind tunnel was operated with a liquid insecticide flow rate of 0.4 mL/min and a nozzle air pressure of 2 PSIG to achieve the largest droplet size under the conditions of this test and to minimize FP waste. To achieve particle-free air, lab air flowed through a five-stage desiccant air dryer (Speedaire-2YNL6, Philadelphia, PA, USA) prior to entering the nozzle [7]. Three replicate mosquito cages (11–18 mosquitoes/cage) per population and FP were each exposed to aerosolized FP droplets for 10 s in the wind tunnel using established methods [7]. Additional replicate mosquito cages were exposed to air in the wind tunnel for 10 s as negative controls or with FPs for analysis of droplet distribution and spread.

### 2.4. Formulated Products

Aerosol testing and mosquito exposures were conducted with the following FPs: (1) Biomist^®^ 3 + 15 (3% permethrin, 15% piperonyl butoxide [PBO], 82% other ingredients, including petroleum distillate [oil-based]), (2) Duet^®^ (1% prallethrin, 5% sumethrin, 5% PBO, 89% other ingredients, including petroleum distillate [oil-based]), (3) AquaDuet^®^ (1% prallethrin, 5% sumethrin, 5% PBO, 89% other ingredients [water-based]), and (4) ReMoa Tri^®^ (4% fenpropathrin, 1.5% abamectin, 0.33% octanoic acid, 0.33% nonanoic acid, 0.33% decanoic acid, 93.51% other ingredients [oil-based]).

### 2.5. Droplet Spread and Number

To determine droplet spread on mosquitoes, riboflavin (fluorescent dye) was mixed with each FP (1 g/L; dye:product ratio) (Biomist^®^, Duet^®^, AquaDuet^®^, ReMoa Tri^®^) before being applied to two replicate mosquito cages (11–18 mosquitoes/cage) per population and FP. Fluorescent dyes are a commonly used method to visualize FP droplets [23]. An additional three cages were used for mortality assessments. A VisiLED ultraviolet (UV)/bright field ring light S80-55 with VisiLED MC 1100 controller to adjust light settings (Schott, Duryea, PA, USA) was used and attached to an SZ61 dissecting microscope (Olympus, Center Valley, PA, USA). The ring light emits UVA light wavelengths of 340–420 nm. Here, the controller was set to four-segment mode to improve contrast and the rotary knob allowed for seamless switching between bright field and UV modes, following manufacturer specifications. The brightness level was set to 10 during mosquito droplet assessment for optimum visualization. The number of droplets was counted for mosquitoes in two replicate cages per population and FP. Total droplets were recorded for the following body parts for each mosquito population and treatment group: proboscis, antennae, head, thorax, wings, legs, and abdomen. Total droplets were also tabulated for each individual mosquito.

### 2.6. Statistical Analyses

Chi-square tests (*p* < 0.05) were used to determine differences in mortality rates between FPs and mosquito populations (SAS Institute, version 9.4, Cary, NC, USA). Mosquito cages were used as the unit of replication using per-cage mortality proportions (i.e., number dead/total mosquitoes per cage). Analysis of variance (*p* < 0.05) was used to determine differences in means of droplet counts between mosquitoes in different treatment groups. Normality was assessed using Kolmogorov–Smirnoff tests and data were transformed prior to statistical analyses, if necessary. If significant differences were observed in the mean, Duncan means comparison tests were used to determine which means were significantly different (*p* < 0.05). Regression analysis (*p* < 0.05) was used to predict if total droplets on mosquito bodies were related to mosquito mortality rates for different FPs.

## 3. Results

Aerosol characterization and mosquito exposures were conducted with the following FPs: (1) Biomist^®^, (2) Duet^®^, (3) AquaDuet^®^, and (4) ReMoa Tri^®^. Droplet sizes (MMD) of FPs were determined by APS prior to wind tunnel mosquito assessments (Biomist^®^ = 4.70 µm, Duet^®^ = 4.40 µm, AquaDuet^®^ = 4.37 µm, ReMoa Tri^®^ = 4.60 µm).

**Figure 2:**
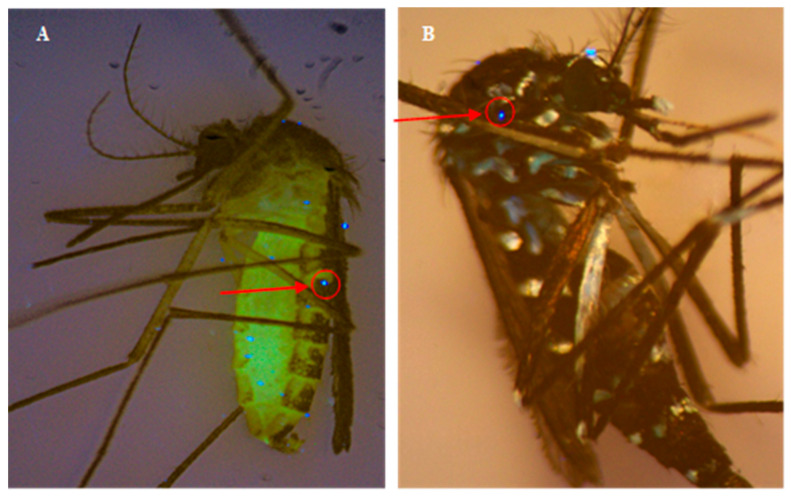
Examples of microscope images of FP with fluorescent dye (riboflavin) on (**A**) *Culex* and (**B**) *Aedes* mosquitoes under UV light filter after wind tunnel exposure. Red arrow/circle indicates example of fluorescent droplet on mosquito body.

Fluorescent droplets were visualized on mosquitoes exposed in the wind tunnel using UV filter and microscopy and tabulated for the four different FPs and mosquito populations (Figure 2, Table 2). The total number of droplets on an individual mosquito body ranged from 0 to 20. No significant differences (*p* > 0.05) were observed in log-transformed total droplet counts between FPs and mosquito populations.

Significant differences were observed in droplet counts between body parts and this varied by FP and mosquito population. Abdomen and legs consistently showed the highest number of droplets in this experiment. Body parts with significantly highest droplet counts for each FP (Duncan statistical comparison) included the following: Biomist^®^ (*p* = 0.001): Abdomen and legs; Duet^®^ (*p* < 0.0001): Abdomen, legs, thorax, and wings; AquaDuet^®^ (*p* = 0.005): Abdomen; and ReMoa Tri^®^ (*p* = 0.001): Abdomen, wings, and legs.

### Mosquito Mortality

**Figure 3:**
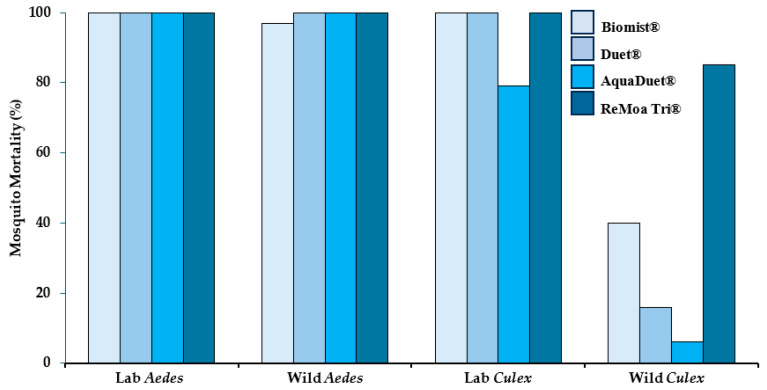
Mosquito mortality 36 h post-exposure in the wind tunnel to insecticide formulated products Biomist^®^, AquaDuet^®^, and Duet^®^ and 48 h post-exposure for ReMoa Tri^®^. Four different mosquito populations were exposed to each product in the wind tunnel for 10 s before being transferred to clean cages for mortality assessment.

Figure 3 shows mortality (36 or 48 h) after exposure to FPs in the wind tunnel to Biomist^®^, Duet^®^, AquaDuet^®^, and ReMoa Tri^®^. No mortality was observed in the control groups and 16–100% mortality was observed for mosquitoes exposed to FP. The lowest mortality rates were observed in the *Culex* wild population: Biomist^®^ (43% mortality), Duet^®^ (16% mortality), AquaDuet^®^ (6% mortality), and ReMoa Tri^®^ (86% mortality). For the other three mosquito populations (*Aedes* lab, *Aedes* wild, *Culex* lab), most mortality rates were > 97%, showing susceptibility. The exception to this was the *Culex* lab colony showing 79% mortality when exposed to AquaDuet^®^.

Regression analyses showed that log-transformed values for total droplet count on mosquitoes (subset of cages characterized) were significantly related to mosquito mortality for Biomist^®^ (df = 1,49, *F* = 11.54, *p* = 0.001, *R*^2^ = 0.191) and ReMoa Tri^®^ (df = 1,57, *F* = 10.66, *p* = 0.002, *R*^2^ = 0.158), but not for AquaDuet^®^ (df = 1,50, *F* = 2.56, *p* = 0.116, *R*^2^ = 0.049) or Duet^®^ (df = 1,52, *F* = 0.88, *p* = 0.352, *R*^2^ = 0.017).

## 4. Discussion

No significant differences were observed in the droplet counts on mosquitoes exposed to different FPs in the wind tunnel. Wind tunnel experiments are designed to show whether the FP causes mosquito mortality [10]. We know how much FP was delivered to each cage of mosquitoes and how many droplets hit each mosquito in the cage in the wind tunnel under the conditions of this test. Further studies will compare mortality rates of caged mosquito populations between wind tunnel and field trial assessments. Since droplets are screened when passing through the mesh mosquito cage, mortality rates of uncaged mosquitoes would likely be different [9,24]. For example, in a field setting, droplets are carried from the ULV machine nozzle source and become dispersed throughout the environment, depending on distance from the spray line, wind, and other factors. In some cases, free flying mosquitoes may experience higher mortality than caged mosquitoes since the droplets are not screened [24]; however, in other cases, wind may disperse droplets to such an extent that not many droplets impinge on mosquitoes. Another study showing no correlation between volume (i.e., droplet size, dose) and the number of FP droplets indicates that several small droplets might have the same effect as fewer large droplets [11]. This can be considered when evaluating technical differences in the application of FPs via topical, wind tunnel, field trial, or other methods. The wind tunnel model used here is a closed system where mosquito cages are positioned 18 cm from the aerosolized FP and droplets are ensured to impinge on mosquito bodies. During field applications of FPs using a ULV machine, droplets are more dispersed, spread by wind throughout the atmosphere across a large area, and are expected to impinge on mosquitoes that are actively flying through the spray.

In wind tunnel experiments, the mean of the total number of droplets per mosquito is related to mosquito mortality in the Biomist^®^ and ReMoa Tri^®^ group, but not the Duet^®^ or AquaDuet^®^ group. Regression models show low explanatory power; hence, under the conditions of this test, droplet counts on mosquito bodies alone do not explain mortality and the interaction of droplet counts with other factors should also be considered. Another study is underway to compare droplet counts on mosquitoes in a field trial to the wind tunnel. An IR study using the topical insecticide application technique showed that FPs (Aqualuer 20–20, Fyfanon) consistently applied to mosquitoes on body parts such as the head, thorax, and abdomen (but not the wings, legs, or proboscis) showed no differences in mortality [25]. The same study diluted each FP 1000-fold with BVA-13 oil to deliver (via syringe to chilled mosquito) a 267 µm droplet that was expected to contain the same amount of insecticide as in a 27 µm droplet of undiluted FP that would be expected to hit a mosquito during a ULV field treatment. Mortality was assessed after 24 h [25]. However, since the 267 µm droplet size in the aforementioned study exceeds the usual size of ULV droplets and our wind tunnel or field trial droplets, no direct comparisons can be made. Two different topical insecticide method development studies described applying (via syringe to chilled mosquitoes) one 0.5 µL drop of each AI/acetone mixture to the ventral or dorsal thorax of each mosquito and assessing mortality after 24 h [26,27]. It should be noted that mosquitoes in the field absorb residual insecticides through their legs while contacting surfaces or via any part of their bodies when flying through an aerosolized ULV treatment [11]. Hence, the topical application of a droplet of AI to the ventral thorax is a starting point for measuring IR in a technical sense rather than being comparable to field applications of FPs [26]. This is a similar limitation to the CDC bottle bioassay that evaluates IR to residual AI, but not aerosolized FP [8,14,26].

Another study showed 85% mortality (resistant) for wild *Cx. quinquefasciatus* after field trial exposure to ReMoa Tri^®^ [18]. *Aedes albopictus* exposed to Duet^®^ in another field trial showed higher mortality (80%) in front yards compared to back yards (56%), although no differences were observed in droplet density between front and back yards [28]. Here, wild *Ae. albopictus* showed 100% mortality to Duet^®^ after exposure in the wind tunnel. Resistant mosquitoes should be used in IR assessments to approximate the real world as resistance is increasing globally [19]. Others have also shown lower droplet density with distance from the field trial spray line that can be impacted by high or gusty wind conditions [29]. Under the conditions of this test, when the wind tunnel experiment resulted in a relatively low mosquito mortality rate (<90%), this indicated FP resistance, following CDC guidelines. In these situations, a field trial would likely not achieve good results and may not even be necessary. On the other hand, when the wind tunnel experiment gives a nearly perfect mortality rate, a field trial can be used to confirm the result.

### Limitations and Future Work

The current study utilizes an APS to measure MMD rather than a DC-IV device, which is used in the field to adjust ULV atomizer settings and achieve targeted aerosol characteristics. The air flow rate and droplet counts (drops/mm^2^) used in wind tunnel experiments here are lower than wind speeds and droplet counts observed in field trials; hence, higher air flow rates and liquid FP feed rates are being used in ongoing experiments to approximate field conditions. Here, fluorescent droplet counts and spread on mosquito bodies exposed to FPs in the wind tunnel were analyzed and this work will be expanded in future work to include a comparison of droplets analyzed on mosquito bodies exposed during field trials. Mortality was recorded at 36 h or 48 h, and this depended on the mode of action of the product being tested. In the future, a uniform end point will be used for consistency. In future studies, randomized run order and/or blinding during mortality assessments will be considered as this is a common practice in toxicology assays. A new wind tunnel design is being developed with further improvements (e.g., HEPA-filtered air, wind speed approximating field conditions) to decrease cost, simplify the design, and increase ease of use. Testing of additional FPs (e.g., ground-based ULV treatments, aerial treatments, oil- and water-based) and mosquito populations from NC and other MCPs is underway.

## 5. Conclusions

The wind tunnel described here has the potential to directly benefit MCPs and/or other agencies for the rapid assessment of FP efficacy. Insecticide testing could be provided as a service for small- to medium-scale programs unable to conduct field trials or for those interested in evaluating certain FPs to inform purchasing decisions. The wind tunnel can be used as a screening step before field trials or as a proxy if/when field trials are not possible. For example, if target mosquitoes are found to be resistant to a chosen FP in the wind tunnel where droplets are guaranteed to impinge on mosquitoes, there is likely no need to conduct a field trial on that FP for that population. Alternatively, if mosquitoes are susceptible to the FP in the wind tunnel, recommendations would be made to MCPs to use that FP for mosquito control for that mosquito population and/or conduct further testing via field trials. This seasonally collected information could empower MCPs to analyze long term IR trends and target mosquitoes with the most effective FP each year.

Additional studies are needed to improve the operational use of IR data in the field [8,13,30]. Surveys of US MCPs showed that 84% (in 2017) and 74% (in 2023) lack the capacity to conduct insecticide testing themselves [31,32]. Hence, it would be useful for MCPs to have a tool for routinely assessing FPs to inform operational decisions [15]. Topical applications can also assess IR to AIs and FPs but are labor-intensive. A wind tunnel could help MCPs bridge the gap between the CDC bottle bioassay that tests AIs and the operational decisions for using FPs.

## Figures and Tables

**Table 1:** Mosquito populations used in wind tunnel assessments. All wild populations were collected in North Carolina.

	Generation	Location	Nickname
*Ae. albopictus*	>50	Orleans Parish, Louisiana	*Aedes* lab
*Ae. albopictus*	2	March Drive, Bolivia, NC Brunswick County	*Aedes* wild
*Cx. quinquefasciatus*	>50	Benzon Laboratories	*Culex* lab
*Cx. pipiens/quinquefasciatus*	0	Curry Court, Greenville, NC Pitt County	*Culex* wild

**Table 2:** Droplet counts for FPs on mosquito bodies in wind tunnel. Mean of total droplets per mosquito ± standard error (range of droplets/mosquito).

	*Aedes* Wild	*Aedes* Lab	*Culex* Wild	*Culex* Lab
Biomist^®^	6.91 ± 1.08 (2–13)	8.92 ± 1.08 (3–15)	9.93 ± 0.90 (6–18)	3.15 ± 0.49 (1–8)
Duet^®^	3.29 ± 0.45 (0–6)	10.33 ± 1.48 (5–20)	4.57 ± 0.78 (2–12)	5.00 ± 0.64 (2–11)
AquaDuet^®^	4.22 ± 0.64 (2–8)	8.44 ± 0.88 (2–14)	4.00 ± 0.43 (2–7)	4.08 ± 0.58 (1–8)
ReMoa Tri^®^	5.31 ± 1.02 (1–15)	6.09 ± 1.13 (1–12)	9.67 ± 0.72 (4–16)	8.73 ± 1.06 (3–14)

## Data Availability

The original contributions presented in this study are included in the article. Further inquiries can be directed to the corresponding author.

## Abbreviations

The following abbreviations are used in this manuscript:

WNE	West Nile encephalitis
US	United States
WNV	West Nile virus
IR	Insecticide resistance
MCP	Mosquito control programs
FP	Formulated product
ULV	Ultra-low volume
CDC	Centers for Disease Control and Prevention
AI	Active ingredient
BLAM	Blaustein Atomizing Module
HEPA	High-efficiency particulate air
NC	North Carolina
MMD	Mass median diameter
APS	Aerodynamic Particle Sizer
PBO	Piperonyl butoxide
UV	Ultraviolet
